# Label-Free Flow Multiplex Biosensing via Photonic Crystal Surface Mode Detection

**DOI:** 10.1038/s41598-019-45166-3

**Published:** 2019-06-19

**Authors:** Irina Petrova, Valery Konopsky, Igor Nabiev, Alyona Sukhanova

**Affiliations:** 1 Laboratory of Nano-Bioengineering, National Research Nuclear University MEPhI (Moscow Engineering Physics Institute), Kashirskoye Shosse 31, 115409 Moscow, Russian Federation; 2 Institute of Spectroscopy, Russian Academy of Sciences, Fizicheskaya Str. 5, 108840 Troitsk, Moscow region Russian Federation; 3 Laboratoire de Recherche en Nanosciences (LRN-EA4682), Université de Reims Champagne-Ardenne, 51 rue Cognacq Jay, 51100 Reims, France

**Keywords:** Sensors, Bioconjugate chemistry

## Abstract

Circulating cancer markers are metabolic products found in body fluids of cancer patients, which are specific for a certain type of malignant tumors. Cancer marker detection plays a key role in cancer diagnosis, treatment, and disease monitoring. The growing need for early cancer diagnosis requires quick and sensitive analytical approaches to detection of cancer markers. The approach based on the photonic crystal surface mode (PC SM) detection has been developed as a label-free high-precision biosensing technique. It allows real-time monitoring of molecular and cellular interactions using independent recording of the total internal reflection angle and the excitation angle of the PC surface wave. We used the PC SM technique for simultaneous detection of the ovarian cancer marker cancer antigen 125 and two breast cancer markers, human epidermal growth factor receptor 2 and cancer antigen 15-3. The new assay is based on the real-time flow detection of specific interaction between the antigens and capture antibodies. Its particular advantage is the possibility of multichannel recording with the same chip, which can be used for multiplexed detection of several cancer markers in a single experiment. The developed approach demonstrates high specificity and sensitivity for detection of all three biomarkers.

## Introduction

Cancer is a heterogeneous multifactorial disease that is still one of the main causes of death worldwide. Early diagnosis of cancer is essential for successful outcome of treatment^1^. In addition, constant disease monitoring is necessary during the course of cancer treatment to avoid recurrence. Continuous confirmation of malignancy via biopsy is not desirable being invasive and risky for patient. Therefore, a search for tumor biomarkers and for novel approaches to their detection is one of the major research goals for the foreseeable future of cancer diagnosis. A tumor biomarker is a substance abnormally expressed in tumor tissue compared with normal tissues. Tumor markers can be detected in malign tissues or, in the case of circulating tumor markers, in body fluids, e.g., blood serum. The latter choice of the diagnostic samples is preferable, being simpler and less invasive. Of particular interest are studies at the protein level, because they directly reflect the cancer-related changes in protein metabolism and function. Bearing this in mind, we present a novel label-free approach to cancer marker detection in blood serum samples.

At present, multiple diagnostic tools for tumor marker detection in liquid samples are being developed. Traditional methods, such as Western blot and ELISA, are easy and cheap but low-throughput and time-consuming. They are suitable for the use in clinical practice but require sample preparation and additional enzymatic or fluorescent labeling, which increases the material and labor cost of the analysis.

Recently, electrochemical immunoassay^2,3^, chemiluminescence imaging immunoassays^4^, fluorescence-labeled dielectrophoresis^5^, photonic suspension array^6^, and label-free affibody-based electrochemical detection approaches have been developed. Label-free affibody-based electrochemical detection is an elegant and sensitive method, but it lacks the capacity for multiplexing^7^. A major advantage of other techniques is multiplexing, that is, the possibility of simultaneous detection of several markers. However, unlike traditional methods, they are quite expensive, difficult to interpret and, hence, unsuitable for clinical practice. Most of these methods require additional labeling for detection as well.

A very advantageous approach is offered by surface plasmon resonance (SPR) technique, which is based on the excitation of the surface plasmon polaritons along a metal/dielectric interface by incident light with a wave-vector matching that of the SPR^8^. This label-free method is sensitive, rapid and does not require sample preparation. In this approach, a thin gold film is coated with marker-specific antibodies, and mass transfer due to association of soluble antigen with marker-specific antibodies on the surface of the film is recorded as a change in the refractive index.

Here, we present a novel approach based on simultaneous quantitative detection of several human serum tumor markers using photonic crystal surface modes (PC SMs) registration approach. PCs are materials characterized by periodic modulation of their refraction indices (RIs) on the scale of the wavelength of light. In recent years, many different types of PC-based optical biosensors have been proposed (see^9^ for the latest review). We exploit a biosensor based on a 1D PC, which is a simple periodic multilayer stack. Such planar 1D PC chips are very easy to produce using the standard equipment for multilayer coating, while 2D and 3D photonic waveguides require complex nanofabrication facilities that impede mass production. In addition, 1D PC chips can be reused many times after standard UV or plasma cleaning.

Such photonic multilayer structures have SMs that support long-range propagation of optical surface-bound waves along their outer border. PC SMs are used in numerous applications in the field of optical sensors^10–14^. The PC SM detection sensors have been reported to have a better sensitivity than SPR-based sensors^10,12^. The detection of both polarizations is possible in the PC SM approach; it allows separate recording of the thickness of the adlayer and the bulk RI, so that the specific signal reflects the association of the analyte to the sensor surface regardless of the bulk RI variations. In addition, a major advantage of a PC-chip is easy regeneration of its surface, which can be plasma-cleaned and tagged with the antibody molecules specific to another type of receptor. Therefore, custom chips can be prepared for analysis of different specific receptors after consequential regenerations of the surface of the same chip.

## Materials and Methods

### Reagents

Ethanol, 99.5%, ACS reagent, absolute (Acros Organics, AC615090010); acetone, 99.8%, for HPLC (Acros Organics, 268310010); (3-aminopropyl)triethoxysilane (APTES), 99% (Sigma-Aldrich, 440140); glutaric aldehyde, grade I, 25% in H_2_O, specially purified for use as an electron microscopy fixative (Sigma-Aldrich, G5882); sodium phosphate dibasic heptahydrate, ACS reagent, 98.0–102.0% (Sigma-Aldrich, S9390); sodium phosphate monobasic monohydrate, ACS reagent, ≥98% (Sigma-Aldrich, S9638); phosphate buffered saline (PBS), tablet (Sigma-Aldrich, P4417); protein A, United States Pharmacopeia (USP) Reference Standard (Sigma-Aldrich, 1578805); CanAg CA 125 EIA kit (anti-CA 125 antibodies and calibrator) (Fujirebio, 400-10); CanAg CA 15-3 EIA kit (anti-CA 15-3 antibodies and calibrator) (Fujirebio, 200-10); Herceptin (Herticad®, BIOCAD); HER2 Platinum ELISA kit (eBioscience, BMS207); bovine serum albumin (BSA), lyophilized powder, crystallized, ≥98.0% (GE) (Sigma-Aldrich, 05470).

### Photonic crystal surface wave detection system

**Figure 1:**
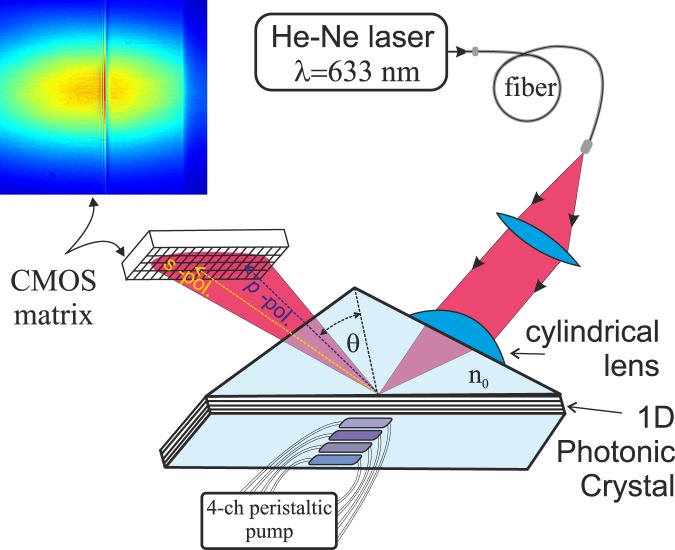
A sketch of the EVA biosensor based on angle interrogation of a photonic crystal surface mode (PC SM). A circular-polarized laser beam from a stabilized He–Ne laser is sent to the sensor surface through a polarization-maintaining fiber cable. The beam is focused by a cylindrical lens in such a way that the excitation angle of one s-polarized PC surface mode (existing in this 1D PC) and TIR angle (in p-polarization) are contained in the convergence angle of the beam. The reflected angular profile (see the color inset) is recorded by the CMOS matrix. The one-dimensional spatial selectivity of this setup allows simultaneous recording of biochemical reactions in four fluid channels.

EVA 2.0, a PC SM biosensor with an independent recording of the critical angle of the liquid, was employed in this study^10,13,14^. To improve the quality of the beam profile, a circular-polarized laser beam from a stabilized He–Ne laser (λ = 632.8 nm) is sent to the sensor surface through a polarization-maintaining fiber cable (Fig. 1). After reflection from the sensor surface, the reflection profile contains information about the PC SM excitation angle (ρ_SM_, in s-polarization) and about the critical TIR angle of the liquid (ρ_TIR_, in p-polarization), which immediately provides us with the RI of the liquid^14^.

The 1D PC structure “**substrate/(LH)**^**3**^**L′/water**”, where L is a SiO_2_ layer with a thickness of d_1_ = 233.1 nm, H is a Ta_2_O_5_ layer with a thickness of d_2_ = 72.5 nm, and L′ is a SiO2 layer with a thickness of d_3_ = 449.5 nm is used in experiments. The seven-layered SiO_2_/Ta_2_O_5_ structure (with SiO_2_ layers at the beginning and at the end of the multilayered structure) is deposited by magnetron sputtering. The prism and the glass plate substrate are made from BK-7 glass. The RIs of the substrate, SiO_2_, Ta_2_O_5_, and water at λ = 632.8 nm are n_0_ = 1.515, n_1_ = n_3_ = 1.47, n_2_ = 2.07, and n_e_ = 1.332, respectively.

A cylindrical lens is used to focus the laser beam on the surface of a PC chip; therefore, one-dimensional spatial selectivity takes place in this biosensor. This allows us to record several immunoassays on the surface simultaneously. In the present study, we used a four-channel fluid cell pumped by a four-channel peristaltic pump and simultaneously detected four different analytes.

**Figure 2:**
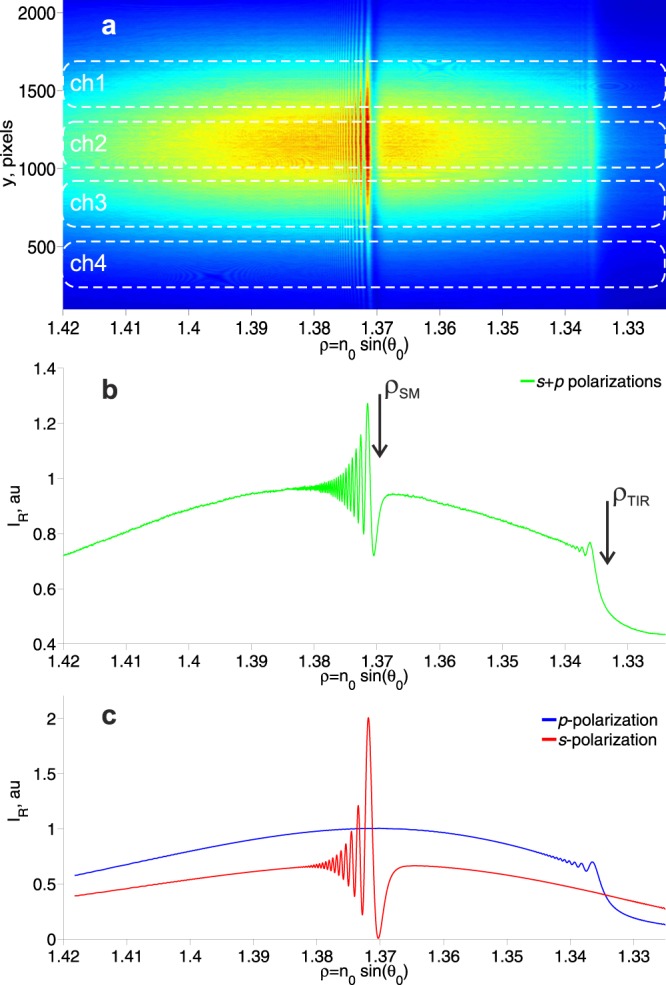
The reflected profiles containing data on the excitation angles of the surface mode (SM) resonance and the total internal reflection (TIR) angle. (**a**) The experimental data recorded by means of a CMOS matrix. The boundaries of the four fluid channels are indicated by dotted white lines. (**b**) Experimental data on the sum of all horizontal lines of the matrix. Arrows indicate the angle of excitation of the SM (ρ_SM_ = n_0_ sin(θ_SM_)) and the angle of the TIR (ρ_TIR_ = n_0_ sin(θ_TIR_)). **(c)** Theoretical calculation of the reflection profiles for s- and p-polarizations. The fine interference structure near ρ_SM_ and ρ_TIR_ is explained in detail in^15^.

A typical reflected profile recorded by a CMOS matrix is shown in Fig. 2a. The sum of all the horizontal lines of the matrix is shown in Fig. 2b (note that, when the signal for a particular channel is recorded, only the lines from this channel are summed).

These angular characteristics contain information about the angle of excitation of the SM and about the angle of the TIR (ρ_i_ = n_0_ sin(θ_i_)). The theoretical reflection profiles for this multilayer structure are shown in Fig. 2c. One can see the agreement between the experimental (Fig. 2b) and the theoretical (Fig. 2c) profiles. The appearance of a fine interference structure near ρ_SM_ and ρ_TIR_ is explained in detail in^15^. This interference profile increases the accuracy of angular resonance detection.

### Biosensor chip activation and functionalization

Before use, each side of the SiO_2_-covered PC-chip was cleaned in a UV purifier for 30 min and then sonicated in acetone, ethanol, and thrice in milliQ water for 5 min before drying at 70 °C. The cleaned PC chips were incubated in a 1% APTES solution in acetone overnight to functionalize them with NH_2_ groups and then baked for 90 min at 120 °C. The resultant amine-coated chips could be stored for a long time. Before measurement, a chip was treated with 2.5% glutaric aldehyde in 20 mM sodium phosphate buffer, pH 7.4, for 30 min under gentle shaking. Note that we did not use dextran or another polymer as a supporting scaffold to bind protein molecules.

The activated chip was placed into a flow cell; inlet and outlet tubes were mounted. A peristaltic pump was used to ensure a constant flow through the cell at a flow rate of 30 μL/min.

To conjugate capture antibodies with chip surface, a 50 μg/mL protein A solution in PBS was run through the flow cell until signal stabilization; then, the surface was rinsed with PBS for 2 min. After the formation of a protein A monolayer, it was used to tag commercially available marker-specific antibodies. Protein A ensured a uniform orientation of IgG molecules after their binding. After that, 2.5–50.0 μg/mL IgG solution in PBS was pumped through the flow cell until signal stabilization. Then, the unconjugated surface of the chip was blocked with 1% BSA in PBS. The flow cell was rinsed with PBS until the signal reached a plateau. The sample solution was pumped through the flow cell at a rate of 30 μL/min.

When a four-channel flow cell was used, the first channel was covered with 1% BSA instead of capture antibodies and served as a negative control. The other three channels were functionalized with monoclonal antibodies against ovarian cancer marker cancer antigen 125 (CA125) and two breast cancer markers, human epidermal growth factor receptor 2 (HER2) and cancer antigen 15-3 (CA15-3).

## Results and Discussion

Three tumor markers selected for detection are markers of female-specific cancers. CA 125, also known as mucin 16 or MUC16, is a large membrane glycoprotein belonging to the mucin family^16,17^. It is well known that mucins are overexpressed and aberrantly glycosylated in many adenocarcinomas, including breast, lung, gastric, colorectal, pancreatic, cervical, and ovarian cancers. CA 125 has been recognized by FDA as an ovarian cancer biomarker. CA 125 has been shown to be a potential NK-cell response inhibitor *in vitro*^16^. In addition, the role of CA 125 in cellular adhesion and, hence, in metastasizing has been described^17^. Serum levels of CA 125 greater than 35 U/mL are diagnostic for the recurrence of epithelial ovarian cancer under specific clinical conditions. Being a large transmembrane glycoprotein (the CA 125 antigen is actually a heterogeneous mixture of glycoproteins with a molecular weight range of 200–1000 kDa^18^), CA 125 is a good candidate for PC SM detection, because this method relies on detection of mass transfer.

HER2 is a membrane tyrosine kinase and, when activated, affects cell proliferation and survival^19^. HER2 gene amplification and/or protein overexpression has been identified in 10–34% of invasive breast cancers. In a great majority of cases, abnormalities in HER2 expression at the gene or protein level are associated with an adverse prognosis in both lymph-node-negative and lymph-node-positive breast cancers^20^. Healthy individuals have a HER2 concentration in the blood in the range between 2 and 15 ng/mL^21^. The screening of HER2 on tumor biopsies is often used to evaluate whether a patient may successfully respond to therapy with trastuzumab (Herceptin), a monoclonal anti-HER2 antibody.

CA15-3 is a mucinous glycoprotein, one of the products of the Mucin1 (MUC-1) gene. CA 15-3 is found in nearly all epithelial cells, and its overexpression is often associated with colon, breast, ovarian, lung, and pancreatic cancers. Statistically significantly elevated levels of serum CA15-3 and CEA in patients with malignant breast tumors, but not in the patients with benign tumors, have been found^22^. Although malignant tumors show elevated levels of CA15-3 at all stages, the level of CA15-3 is strongly associated with the tumor stage, which means that the level of CA15-3 becomes higher as the breast tumor progresses^23^.

It should be noted that, due to limited specificity and sensitivity of most known biomarkers, a single biomarker is not a prognostic indicator. An elevated level of a single biomarker may indicate different malignant and even benign conditions; on the other hand, some forms of cancer do not demonstrate all of the possible markers. Therefore, multiplexed detection of different proteins is highly important. Here, we have developed a system for simultaneous detection of three tumor biomarkers: two for breast cancer (HER2 and CA 15-3) and one for ovarian cancer (CA 125).

For this purpose, we used a four-channel flow cell for PC SM detection. A glass cuvette with four separate chambers, each with its own inlet and outlet, was pressed against the sensing surface of a multilayered tantalic oxide–silicon oxide photon crystal. The sensing surface for detection of tumor markers in a liquid sample was functionalized with monoclonal antibodies. The affinity and purity of the capture antibodies is highly important for successful detection, because each non-reactive molecule reduces the working surface area of the sensor chip. The signal amplitude is in direct proportion to the number of binding sites on the sensing surface. Therefore, before immobilization of antibodies on the surface of PC at their correct and uniform orientation, the surface was covered with Fc-binding protein A. After antibody deposition, the non-functionalized areas of sensor surface were blocked with BSA to avoid nonspecific binding.

In order to assess the specific binding of tumor markers to the functionalized surface of the PC chip, solutions of commercially available antigens in PBS were run through the flow cell at a rate of 30 μL/min. Different concentrations of different tumor markers were used: 10 U/mL for CA125, 50 pg/mL for HER2, and 15 U/mL for CA15-3. Commercially available solutions of tumor markers were used either in the form of calibrators for diagnostic ELISA kits CanAg CA 125 EIA and CanAg CA 15-3 EIA (Fujirebio, Inc.) or in the form of a HER2 control sample for HER2 Platinum ELISA kit BMS207 (eBioscience).

**Figure 3:**
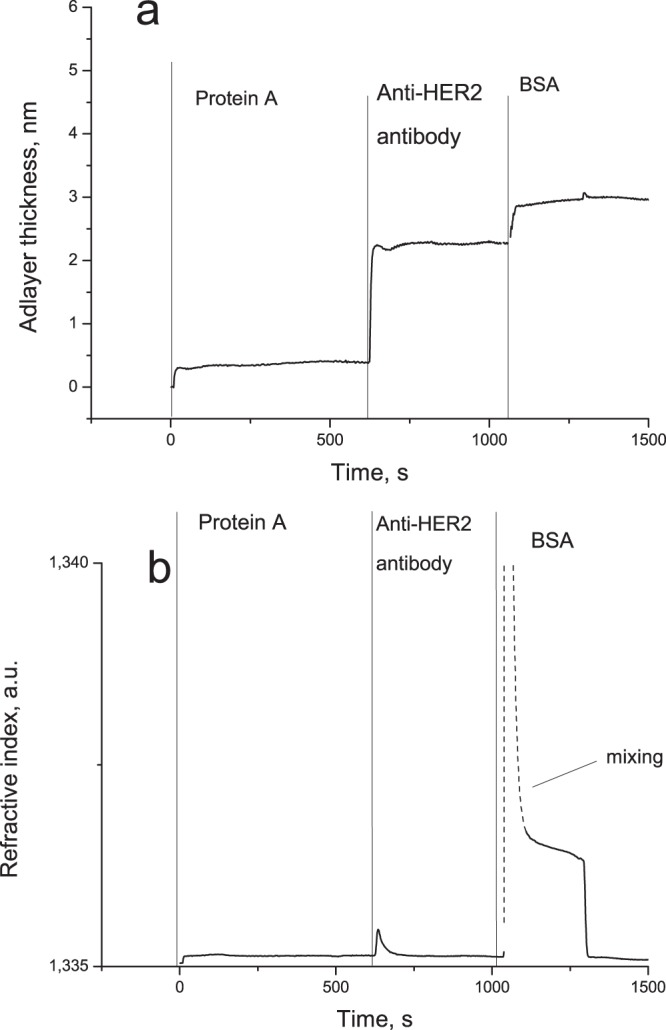
The sensorgram reflecting a typical formation of the sensor surface. **(a)** The adlayer thickness signal; the SiO_2_-based surface of the PC chip is conjugated with protein A via a combination of APTES and glutaric aldehyde; the next step is deposition of IgG antibodies on the protein A layer; after that, BSA is used to block the unreacted surface to avoid nonspecific binding; the resulting chip is ready for the detection of an antigen (HER2, in this case). **(b)** The refractive index signal.

Figure 3 shows the formation of the sensing surface for tumor marker detection. The graph shows the change in the adlayer thickness upon successive binding of protein A with the glutaric aldehyde-treated surface, antibodies with protein A (50 μg/mL herceptin), and BSA with the unreacted surface. After each stage, the flow cell was rinsed with PBS for about 1 min. The resulting curve is similar to an SPR sensorgram and follows the same kinetics^24^. Deposition of a protein layer on the sensor surface causes a rapid increase in adlayer thickness, which reaches plateau after an initial steep phase. The plateau corresponds to the steady state, when the rates of adsorption and desorption are equal, and the transient can be approximated by the Langmuir adsorption model. The subsequent rinsing of the sensor surface with PBS is accompanied by a decline of the adlayer thickness until the system reaches a new equilibrium. The rates of the increase and decrease depend, respectively, on the association and dissociation constants for the pair “soluble ligand (analyte) – surface-bound moiety (receptor)”. The peak on the graph (dashed line) demonstrating the variation of the refractive index signal is caused by the irregular flow in the chamber due to the rapid change in the density as the buffer is replaced with the BSA solution. Once the flow chamber is completely filled with the latter, the refractive index signal returns to normal. Since the adlayer thickness and refractive index are measured separately, artifacts like this do not disturb the adlayer thickness measurement, as can be seen in Fig. 3a.

**Figure 4:**
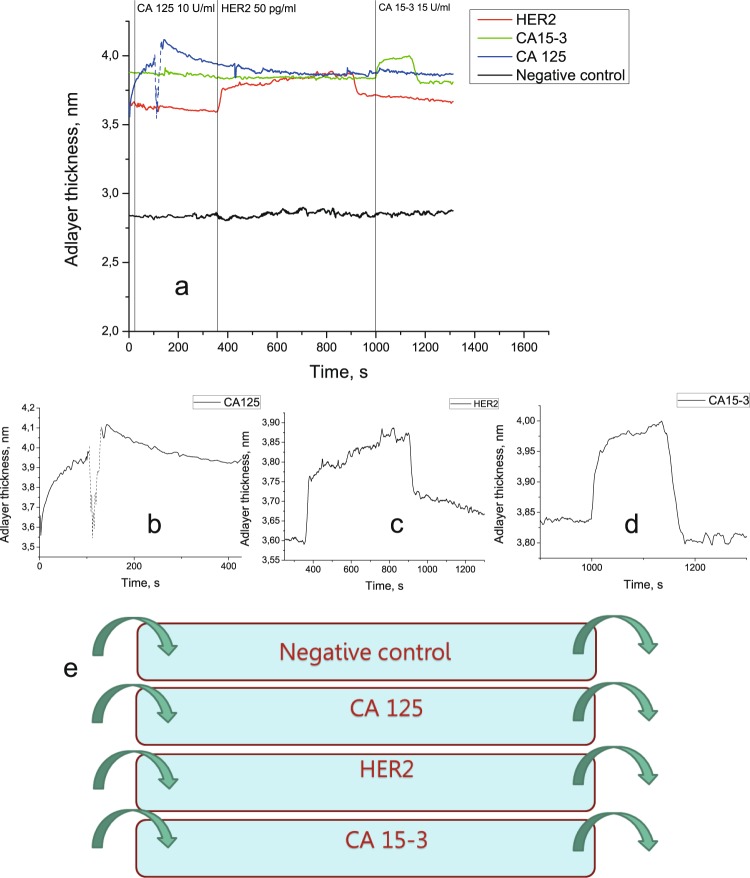
The experiment with four-channel flow cell. **(a)** Parallel detection of three soluble tumor markers in a four-channel flow cell containing antibodies against CA125, HER2, and CA15-3. The dip in the signal corresponding to CA125 detection (dashed line) is caused by an air bubble formation inside of the flow cell. **(b)** A signal corresponding to CA125 detection. **(c)** A signal corresponding to HER2 detection. (**d**) A signal corresponding to CA15-3 detection. **(e)** A schematic diagram of the four-channel flow cell used in the experiment.

In the case of a four-channel flow cell, the solutions in question were run through all four channels in the following order: CA125, HER2, and CA15-3. The flow cell was rinsed with PBS between each two antigen solutions. Figure 4 shows successive detection of three biomarkers in a single flow cell. The absence of cross-reactivity is demonstrated by the lack of a signal from the sample containing a tumor marker in the channels corresponding to other markers. The nonspecific response was within the standard deviation of the signal corresponding to pure PBS.

The mass limit of PC SM detection was predicted earlier to be 70 fg/mm^2^ on the probed spot of the sensing surface^12^. A quantitative detection is highly important for correct evaluation of the tumor biomarker status. It is hard to directly compare the sensitivities in the case of the biomarkers studied, because the CA 125 and CA 15-3 concentrations are measured in arbitrary units, whereas the HER2 concentration, in grams per milliliter.

Nevertheless, the physiologically relevant values for all three biomarkers are known. For CA15-3, a cut-off value of 30 U/mL is used^25^, although some studies have used a cut-off value 60 U/mL^26^. For CA125, values below 35 U/mL are considered normal^18^ (although a value of 16 U/mL was suggested for detection of ovarian cancer recurrence^27^), and for HER2, the normal range is 2–15 ng/mL in the blood^21^. As can be seen, the sensitivity of the developed assay allows the detection of the normal concentrations of all three biomarkers, let alone elevated ones. The next question is the possibility of quantitative detection, which would allow the discrimination between normal and pathological values. For this purpose, the range of linearity should be determined. The linear model is the simplest for analysis, but in the steady-state model, only the initial interval of the concentration–signal curve can be approximated by a linear dependence. In the case of CA 15-3, the linear range is 0–50 U/mL; in the case of CA 125, 0–40 U/mL. For HER2, the detected values are in the concentration range of picograms per milliliter.

**Figure 5:**
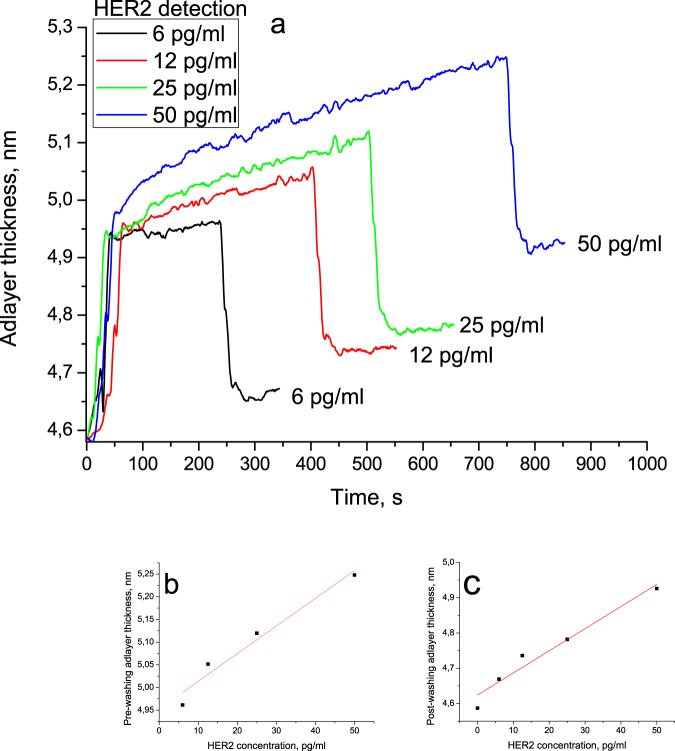
Quantitative detection of HER2 antigen using photonic crystal surface mode detection. (**a**) The dependence of the adlayer thickness increase and decrease on the HER2 concentration; after the start of the HER2 flow, the signal was allowed to reach a plateau (pre-washing adlayer thickness); then, the solution was switched to PBS to begin the washing stage; the washing stage was marked with a rapid decrease in the signal intensity until it reached a new plateau (post-washing adlayer thickness). (**b**) The linear dependence of the pre-washing adlayer thickness on the HER2 concentration. (**c**) The linear dependence of the post-washing adlayer thickness on the HER2 concentration (the value before HER2 binding taken to be the zero point).

To assess the mass sensitivity of the sensor, HER2 was selected for detailed investigations. To assess the range of linearity, several concentrations should be used for analysis. Solutions with concentrations 6, 12, 25, and 50 pg/mL were run through the flow cell. The resulting sensorgrams are shown in Fig. 5. As expected, the sensor response increased with increasing concentration. After the start of the HER2 flow, the signal was allowed to reach a plateau (pre-washing adlayer thickness); then, the solution was switched to PBS to begin the washing stage. The washing stage was marked with a rapid decrease in the signal until a new plateau was reached (post-washing adlayer thickness). The pre-washing thickness linearly depended on the HER2 concentration (R^2^ > 0.95), the same was true for the post-washing thickness. Note that, unlike the conventional SPR protocol, pre-washing thickness was assessed when the signal reached a plateau (the difference between the maximum and the minimum points in a 128-s interval not exceeding 0.03 nm), which took a longer time in the case of higher concentrations. This effect was to be expected because, at higher concentrations, more binding sites should be occupied, so that the equilibration time reflects how long it takes the ‘on’ kinetics to deliver a sufficient amount of target molecules. At lower concentrations, the equilibration process is governed mostly by the ‘off’ kinetics^28^. However, the concentration dependence of the signal values measured at the same moment after beginning of the HER2 run exhibit a linear behavior as well.

Using the pre-washing adlayer thickness linear fit, the limit of detection (LOD) for HER2 as the concentration that corresponds to three standard deviations of the sensor response to a blank sample was calculated to be 620 fg/mL. The obtained value of LOD is well below the values reported for carcinoembryonic antigen (CEA) and pregnancy-associated plasma protein A2 (PAPP-A2) in gold-nanoparticle-enhanced SPR^29,30^, although our approach is label-free and does not require signal enhancement with gold nanoparticles. This may be due to a higher reactivity of anti-HER2 antibodies. The sensitivity of the suggested approach exceeds that of electrochemical immunoassays as well^3^. Moreover, the sensitivity of our method in HER2 detection is better than reported for an impedimetric biosensor employing affibodies LOD values of 6 ng/mL^21^. The minimal detectable concentration for CA125 is 0.55 U/mL, and that for CA15-3 is 1.84 U/mL.

## Conclusions

A label-free photonic crystal surface wave detection biosensor obtained by immobilizing monoclonal antibodies on the optical chip surface for the detection of the CA 125, CA15-3, and HER2 tumor biomarkers has been developed. The new assay allows simultaneous detection of three tumor markers in a single experiment. Highly sensitive detection has been demonstrated for HER2 antigen, with a limit of detection of 620 fg/mL and a linearity range of up to 50 pg/mL. In comparison with the conventional HER2 detection methods, the photonic crystal surface wave detector displays a fast, sensitive, and specific detection of HER2 with a very low detection limit, yielding promising results in terms of clinical applications.

## Data Availability

All data generated or analysed during this study are included in this published article.
